# Ophthalmic Manifestations and Genetics of the Polyglutamine Autosomal Dominant Spinocerebellar Ataxias: A Review

**DOI:** 10.3389/fnins.2020.00892

**Published:** 2020-08-21

**Authors:** Jun Young Park, Kwangsic Joo, Se Joon Woo

**Affiliations:** Department of Ophthalmology, Seoul National University Bundang Hospital, Seoul National University College of Medicine, Seoul, South Korea

**Keywords:** autosomal dominant, spinocerebellar ataxias, polyglutamine, ophthalmic manifestations, genetics

## Abstract

Spinocerebellar ataxia (SCA) is a part of the cerebellar neurodegenerative disease group that is diverse in genetics and phenotypes. It usually shows autosomal dominant inheritance. SCAs, always together with the cerebellar degeneration, may exhibit clinical deficits in brainstem or eye, especially retina or optic nerve. Interestingly, autosomal dominant SCAs share a common genetic mechanism; the length of the glutamine chain is abnormally expanded due to the increase in the cytosine–adenine–guanine (CAG) repeats of the disease causing gene. Studies have suggested that the mutant ataxin induces alteration of protein conformation and abnormal aggregation resulting in nuclear inclusions, and causes cellular loss of photoreceptors through a toxic effect. As a result, these pathologic changes induce a downregulation of genes involved in the phototransduction, development, and differentiation of photoreceptors such as *CRX*, one of the photoreceptor transcription factors. However, the exact mechanism of neuronal degeneration by mutant ataxin restricted to only certain type of neuronal cell including cerebellar Purkinje neurons and photoreceptor is still unclear. The most common SCAs are types 1, 2, 3, 6, 7, and 17 which contain about 80% of autosomal dominant SCA cases. Various aspects of eye movement abnormalities are evident depending on the degree of cerebellar and brainstem degeneration in SCAs. In addition, certain types of SCAs such as SCA7 are characterized by both cerebellar ataxia and visual loss mainly due to retinal degeneration. The severity of the retinopathy can vary from occult macular photoreceptor disruption to extensive retinal atrophy and is correlated with the number of CAG repeats. The value of using optical coherence tomography in conjunction with electrodiagnostic and genetic testing is emphasized as the combination of these tests can provide critical information regarding the etiology, morphological evaluation, and functional significances. Therefore, ophthalmologists need to recognize and differentiate SCAs in order to properly diagnose and evaluate the disease. In this review, we have described and discussed SCAs showing ophthalmic abnormalities with particular attention to their ophthalmic features, neurodegenerative mechanisms, genetics, and future perspectives.

## Introduction

Ataxia, defined as impaired coordination of voluntary muscle movement, is usually caused by cerebellar dysfunction, but it can also be caused by impaired vestibular or proprioceptive afferent inputs to the cerebellum or damage to the spinal cord. Acute etiology including cerebellar infarction, hemorrhage, or infection can induce ataxia, but it can also have an insidious onset with a chronic and slowly progressive clinical course such as inherited neurodegenerative disorders (Kersten et al., 2014; Ashizawa and Xia, 2016).

Spinocerebellar ataxias (SCAs) are one of the inherited progressive neurodegenerative disorders and belong to the phenotypically and genetically diverse group of cerebellar ataxias. Many of the SCAs have an autosomal dominant inheritance pattern. The prevalence of SCAs is known as 1–5 cases per 100,000 persons (Duenas et al., 2006). Each SCA have different clinical manifestations, and mutations in more than 30 distinct genetic loci encoding protein kinases, protein phosphatases, membrane receptors, intracellular ion channels, plasma membrane, and protein with unknown function (Meera et al., 2016). However, the autosomal dominant SCAs mainly have a similar genetic mutation, which the length of the glutamine chain is abnormally expanded due to the increase in the cytosine–adenine–guanine (CAG) trinucleotide repeat of the disease causing gene (Meera et al., 2016). Also, autosomal dominant SCAs share a variety of common characteristics including adult onset of symptoms (most common between 30 and 50 years of age), autosomal dominant inheritance, distinct neural degeneration resulting in disease-specific signs, and intracellular accumulation of amyloid-like aggregates composed of mutant proteins (Zoghbi and Orr, 2000; Duenas et al., 2006). An extended polyglutamine (polyQ) chain in a mutant form of protein induces altered protein conformation and abnormal protein aggregation, resulting in a loss of function of the protein and consequential toxic effect, ultimately leading to cell death. It has been known that glutamate receptor signaling and disrupted calcium homeostasis are involved in this degenerative process (Meera et al., 2016). The protein encoded by the *ataxin* gene is ubiquitously expressed. It has been postulated in that many SCAs also affect the retina and/or optic nerve, and other regions of the central and peripheral nervous systems, which can be one of the explanations by the fact that they share the identical embryological origin (neuroectoderm) (Spina Tensini et al., 2017).

Up to February 2020, approximately 50 genetically different types of SCAs (SCA1–SCA48) are registered (http://www.ncbi.nlm.nih.gov/sites/omim), but the number is expected to increase with the discovery of new genotypes. SCA15 and SCA19 were found to be the same entity as SCA16 and SCA22, respectively. SCA24 is recessively inherited. Approximately 80% of autosomal dominant SCA cases are one of SCA 1, 2, 3, 6, and 7, which are the most common polyQ expansion disorders, and will be the main topics of this article (Lynch and Farmer, 2002; Coarelli et al., 2018). This group of diseases is designated as autosomal dominant cerebellar ataxias (ADCAs) and classified into three types: “ADCA type 1 displays cerebellar ataxia combined with ophthalmoplegia, dementia, extrapyramidal signs, optic atrophy, and amyotrophy. SCA7 is the only type of ADCA type 2 and is characterized by progressive cerebellar ataxia and retinal degeneration. ADCA type 3 is considered pure cerebellar ataxia, in which SCA6 is the most common form, followed by SCA31 (Sun et al., 2016).”

Clinically, SCAs are typically characterized by cerebellar degeneration and cerebellar signs and symptoms, including difficulty with balance, lack of coordination, tremor, and oculomotor abnormalities are common in patients, since the cerebellum is responsible for controlling balance, limb co-ordination, speech, and eye movements (Paulson, 2009). In addition, cerebellar disease can occur isolated or with brainstem or retinal abnormalities. Because the clinical presentation is extremely heterogeneous, combination of several clinical symptoms and signs can aid in the distinction of different SCA type and can useful for differential diagnosis. From this point of view, prominently slow saccade is typical of SCA2, spasticity can be seen in SCA3, pure cerebellar ataxia is hallmark of SCA6, and retinal degeneration is usually observed in SCA7.

From an ophthalmological standpoint, it is an important issue that a diverse range of ophthalmic abnormalities, especially the optic nerve and retina, and eye movement abnormalities have been reported in patients with SCAs. Considerable visual impairment can occur in the affected individuals resulting from these abnormalities. Clinically, visual disturbances may precede other symptoms in certain type of SCAs such as SCA7, which is characterized by cerebellar ataxia with various degree of visual impairment mainly due to retinal degeneration. In addition, subclinical or clinical involvement of the optic nerve and retina has also been observed in SCAs; therefore, ophthalmologists need to suspect whether the ocular involvement exists (Pula et al., 2010; Kersten et al., 2014).

Since the optic nerve head and retina comprise of axons and glia without myelin, they can provide an exclusive approach for investigating neurodegenerative mechanisms (Frohman et al., 2008) and axons of the retinal ganglion cell at the optic nerve are distinguished from other neurons. The emergence of the optical coherence tomography (OCT) enabled more fully recognized clinical evaluation of retina and optic nerve in patients with hereditary neurodegenerative disorders through quantitative analysis of the retinal layers and the optic nerve after rapid and non-invasive cross-sectional imaging. Several parameters measured through OCT are being studied as potential representative biomarkers of neural degeneration in SCAs.

In this review, we have described and summarized the ophthalmic manifestations and genetics of the most common polyQ SCAs (SCA types 1, 2, 3, 6, 7, and 17) that present ophthalmic abnormalities. We have focused on the afferent visual system manifestations, including the retina and optic nerve, and neurodegenerative mechanism and genetics in terms of ocular pathology, and the usefulness of OCT in SCAs. A summary of the ophthalmic manifestations of the polyQ autosomal dominant SCAs outlined in this review is provided in Table 1.

**TABLE 1:** Summary of the common autosomal dominant spinocerebellar ataxias with polyglutamine chain.

**Subtype**	**Gene/location**	**Translated nucleotide repeat**	**Normal repeats (expanded repeats)**	**Protein (major function of protein)**	**Neuropathology/presenting sign**	**Ophthalmic abnormalities**			
						**Retina**	**Optic nerve**	**Oculomotor**	**References**
SCA1	*ATXN1*/ 6p22.3	CAG	6–39 (40–83)	ATXN1 (transcriptional regulation)	Cerebellum, brainstem, and spinal cord/Ataxia, spasticity	Often relatively mild macular degeneration compared to SCA7	Common optic atrophy, significant temporal RNFL thinning	Ophthalmoplegia, saccades hypo/hypermetria, impaired fixation with saccadic intrusion	Pula et al., 2011; Stricker et al., 2011; Lebranchu et al., 2013; Vaclavik et al., 2013; Nishiguchi et al., 2019
SCA2	*ATXN2*/ 12q24.12	CAG	15–32 (33–100)	ATXN2 (RNA repair, ribosomal translation)	Cerebellum, brainstem, substantia nigra, spinal cord and polyneuropathy/Ataxia	Rare pigmentary retinal degeneration in rare variants	Rare optic atrophy, RNFL thinning	Slow saccades	Pogacar et al., 1978; Rufa et al., 2002; Velazquez Perez et al., 2007; Geiner et al., 2008; Pula et al., 2011; Di Fabio et al., 2012; Jensen et al., 2019
SCA3	*ATXN3*/ 14q32.12	CAG	12–42 (45–86)	ATXN3 (deubiquitinase)	Dentate nucleus, basal ganglia, substa ntia nigra, spinal cord and poly neuropathy/Ataxia, spasticity	Not reported, decreased average central macular thickness	Optic atrophy (rare), RNFL thinning (common) with preserved temporal peripapillary sector	Ophthalmoplegia, impaired VOR	Jardim et al., 2001; Pula et al., 2011; Alvarez et al., 2013; Spina Tensini et al., 2017
SCA6	*CACNA1A*/ 19p13.13	CAG	4–18 (19–30)	Voltage-dependent calcium channel subunit alpha-1A (neuronal excitability)	Cerebellum/Pure cerebellar ataxia (onset in the 50s)	Not reported, decreased average central macular thickness	Mostly normal, RNFL thinning (uncommon)	Downbeat, rebound, gaze evoked nystagmus, impaired pursuit and VOR	Pula et al., 2011; Kim et al., 2013; Moscovich et al., 2015
SCA7	*ATXN7*/ 3p14.1	CAG	7–34 (37–200)	ATXN7 (subunit of histone acetyltransferase complexes, transcriptional regulation)	Cerebellum, brainstem, basal ganglia, and retina/Ataxia, dysarthria, visual disturbance	Common diverse degree of retinal degeneration: occult macular dystrophy, cone-rod dystrophy, severe chorioretinal degeneration	Mostly normal, RNFL thinning (uncommon)	Ophthalmoplegia, impaired pursuit, VOR, slow saccades	Aleman et al., 2002; Ahn et al., 2005; Hugosson et al., 2009; Manrique et al., 2009; Campos-Romo et al., 2018; Azevedo et al., 2019; Park et al., 2019
SCA17	*TBP*/ 6q27	CAG/CAA	25–40 (41–66)	TATA box-binding protein (transcriptional initiation factor)	Cerebellum, cerebral cortex, and caudate nuclei/Ataxia, dementia, chorea, psychiatric symptoms, extrapyramidal features	Not reported	Not reported	Saccadic hypometria, increased latency, downbeat, rebound, gaze evoked nystagmus, hyperreflexia of VOR, increased error rate of anti-saccades	Origone et al., 2018; Toyoshima and Takahashi, 2018; Liu et al., 2019; Mariotti et al., 2007; Hübner et al., 2007; Rosini et al., 2020

*SCA, spinocerebellar ataxia; RNFL, retinal nerve fiber layer; VOR, vestibulo-ocular reflex.*

## Spinocerebellar Ataxia Type 1

The *ATXN1* gene, the gene for SCA1, has an expanded CAG repeat encoding a polyQ tract in ataxin-1 localized to chromosome 6p22.3 (Orr et al., 1993). Normal SCA1 alleles have approximately 6–39 repeats. SCA1 pathology manifests in patients with ≥ 40 CAG repeats. The length of the repeat expansion varies between individuals, even within families, with age of onset inversely correlating to the length of the repeat expansion. In the neuronal nucleus, the primary site of pathogenesis, the misfolded ataxin-1 forms abnormal protein aggregations preventing from escaping from the nucleus.

Glutamine-expanded ataxin-1 affects the regulation of gene transcription and RNA splicing. The abnormal interaction between the mutant ataxin-1 protein and other intracellular components results in neuronal dysfunction and degeneration, finally leading to the phenotypic abnormalities observed in SCA1 (Kang and Hong, 2009; Perez Ortiz and Orr, 2018).

Typical SCA1 presents during the third or fourth decades (ranging from 7 to 50 years) with symmetric gait abnormality and progressive ataxia related to cerebellar dysfunction. Many patients are wheelchair-bound by the age of 15–20 years. Pathologic findings are associated with atrophy in Purkinje cells of not only the cerebellum but also the pons and middle cerebellar peduncle. SCA1 is characterized by extracerebellar features and corticospinal tract dysfunctions such as hyperreflexia and spasticity in more than 50% of patients.

SCA1 usually can demonstrate oculomotor disruptions associated with cerebellar connections, such as saccadic pursuit, slowing of saccades, saccadic dysmetria and gaze-evoked and rebound nystagmus (Buttner et al., 1998; Stephen and Schmahmann, 2019). Saccadic latency is generally preserved. Owing to the involvement of brainstem including the parapontine reticular formation, saccades can also become slow and horizontal and vertical ophthalmoparesis may be present in SCA1 (Kim et al., 2013).

Loss of vision in patient with SCA is not frequently reported, but two types of autosomal dominant SCAs, SCA1 and SCA7, are associated with ocular pathology and poor visual outcome resulting from either optic neuropathy or retinopathy. Visual loss in SCA7 is caused by retinal and macular degenerations, whereas that in SCA1 has been initially associated with optic atrophy (Abe et al., 1997). Unlike SCA7, the ophthalmological findings in patients with SCA1 were not correlated with the trinucleotide repeat number of the SCA1 gene. The ophthalmological findings were most highly correlated with the duration of the neuronal disease (Abe et al., 2001).

Despite some studies suggesting that the involvement of retina is not evident in SCA1 (Robitaille et al., 1995), macular dysfunction has been relatively recently reported. Thurtell et al. (2011) reported a case of a genetically proven SCA1 patient with visual loss secondary to rod–cone dystrophy, whose findings are bilateral, progressive visual loss with subtle pigmentation on fundus examination and rod and cone photoreceptor dysfunction on full-field electroretinogram (ERG). A study for macular thickness evaluation using OCT has reported that 7 patients with SCA1 showed significantly reduced average macular thickness within 3 mm of the fovea (Pula et al., 2011). A case report of two patients showing a gradually decreased vision to 6/60, and symmetric hypopigmentation at the macula in both eyes, with OCT demonstrating reduced thickness of the outer nuclear layer, and bilateral hyporeflective foveal cavity corresponding to the disruption of photoreceptor ellipsoid zone has reported, similar to those reported previously in achromatopsia and cone dystrophy (Vaclavik et al., 2013) (Birch et al., 2011). Lebranchu et al. (2013) has introduced 4 members of a SCA1 family showing macular abnormalities from disorganization of the photoreceptor outer segement layer, abnormal foveal outer segment cavitation to bilateral macular atrophy. Nishiguchi et al. have reported that central macular thinning and disruption of the ellipsoid zone on OCT and reduced amplitude in the localized foveal area on multifocal ERG with normal response on full-field ERG, similar to occult macular dystrophy, may result in a significant visual loss in five consecutive SCA1 patients with subtle funduscopic alterations. They also asserted that OCT and multifocal ERG could help to reliably detect these macular pathology associated with SCA1 (Nishiguchi et al., 2019). Color vision abnormalities may present in some SCA1 patients with photoreceptor ellipsoid zone disruption (Oertel et al., 2020).

Recently, various degrees of macular degeneration have been thought as another important cause of visual loss as well as optic nerve dysfunction in SCA1 patients (Lebranchu et al., 2013; Vaclavik et al., 2013). Interestingly, the macular pathology could not be clearly found through funduscopic examination in some patients, which can imply the importance of obtaining OCT and multifocal ERG in SCA1 patients, particularly in those with reduced visual acuity. The findings extend the range of ophthalmologic phenotypes and provide important information to assist the management of families in whom SCA1 is suspected. Pathophysiology for macular pathology in SCA1 is barely understood. Probably, an extended polyQ chain of ataxin-1 induces abnormal protein aggregation, and aberrant interaction between the mutant ataxin-1 protein and other intracellular components, resulting in substantial toxic effect and a loss of function of ataxin-1 as transcriptional regulation, ultimately leading to cell loss. Fernandez-Funez et al. (2000) reported that inducing the expression of polyQ expanded ATXN1 in retina of drosophila subsequently developed a progressive loss of retinal cell bodies and axonal projections. CAG-repeat expansion on *SCA1* may have a possibility to affect nearby multiple genes such as the genes peripherin-2, which is essential for photoreceptor formation and maintenance, and guanylate cyclase activator 1a, a causal gene for cone dystrophy, located in 6p21.1 (Conley et al., 2019) (Manes et al., 2017).

Optic nerve atrophy has been reported as a main cause of visual loss in SCA1 patients (Abe et al., 1997). The major part of SCA1 patients demonstrated abnormalities of both latency and amplitudes of visual evoked potentials, which indicate to evaluate subclinical involvement of the visual pathway (Perretti et al., 1996; Schöls et al., 2008). OCT showed diffuse RNFL thinning in nine patients with SCA1, with the temporal region being most severely affected (Stricker et al., 2011), suggesting that the papillomacular bundle is affected due to optic nerve damage (Stricker et al., 2011). In late stage disease, RNFL atrophy could lead to ganglion cell degeneration and consecutively to macular volume reduction. A recent case series by Nishiguchi et al. reported temporal RNFL thinning (Nishiguchi et al., 2019). Oertel et al. has reported that peripapillary retinal nerve fiber layer thickness and combined ganglion cell and inner plexiform layer volume as markers of optic atrophy measured by OCT were reduced in 20 patients with SCA1 compared with healthy controls (Oertel et al., 2020).

Mechanisms of optic nerve atrophy and progressive RNFL degeneration were not clearly identified in SCA1. It was postulated that nuclear and cytosolic abnormal protein aggregates induce abnormal interactions leading to transcriptional dysregulation, which finally impact on RNFL neuroaxonal damage as part of a central nervous system degeneration similar to neurodegeneration of cerebellar Purkinje neurons and retinal photoreceptors (Doss et al., 2014) (Kang and Hong, 2009). It was also believed that antioxidant mechanisms such as reduced Cu/Zn-superoxide dismutase activity with subsequent accumulation of reactive oxygen species affect the disease process (Kim et al., 2003).

## Spinocerebellar Ataxia Type 2

Genetically, SCA2 is induced by a pathological expansion of the CAG trinucleotide repeats in the coding region of the *ATXN2* gene resided on chromosome 12q24.1 (Gispert et al., 1993). Generally, SCA2 have expansions of 33–100 repeats, but the rare infantile form results from expansions of 100 to 500 repeats in the defective gene (Fernandez et al., 2000).

Ataxin-2 is a 140-kDa cytoplasmic protein, product of the *ATXN2* gene, localized at the rough endoplasmic reticulum. The abnormal ataxin-2 produces a cytosolic protein that is implicated in cytoplasmic RNA-related functions and results in abnormal aggregation, oxidative stress, disturbed cell signaling, dysregulation of calcium homeostasis, abnormal autophagy, and impaired DNA processing, all presumed to be involved in SCA2 molecular pathogenesis (Amarante et al., 2017).

SCA2 is the second most common SCA and shows abnormalities in both cerebellar and brainstem functions. The first clinical presentation demonstrated in the majority of SCA2 patients is a gait dysfunction. Although the main pathological characteristic is olivopontocerebellar atrophy as well as in SCA1, dopaminergic neurons in the substantia nigra and nigrostriatal pathways are more severely affected (Seidel et al., 2012). SCA2 patients can also have progressive cerebellar ataxia, substantial slowing of saccadic velocities, upper motor neuron signs, postural and action tremor, parkinsonism, and polyneuropathy. Some SCA2 patients might have lower cranial dystonia, especially jaw and tongue, as a clinical feature (Markovic et al., 2016).

In SCA2, similar to other polyQ diseases, the age of onset is greatly correlated with the number of repeats of CAG trinucleotide. The onset of disease symptoms usually occurs in the early 30s in SCA2 (Amarante et al., 2017). There is a severe congenital onset variant showing pigmentary retinopathy, infantile spasms, and infantile mortality in part of SCA2 if number of CAG repeat occurs over 200 (Pula et al., 2010).

Approximately 90% of SCA2 patients can have severe slowing of ocular movements and reduced saccade velocity associated with the impairment of excitatory burst neurons in the paramedian pontine reticular formation (Jensen et al., 2019). A velocity reduction depends on the length of the CAG repeat expansions: the longer polyQ expansion, the greater velocity reduction (Velázquez-Pérez et al., 2004; Velazquez Perez et al., 2007). Saccadic slowing was present even in the early stage of SCA2, can insidiously progress, eventually leading to ophthalmoplegia in late stage (Velázquez-Pérez et al., 2009; Stephen and Schmahmann, 2019). It has been reported a saccadic pursuits, nystagmus, saccadic hypometria, prolonged latency and increased rate of directional errors of anti-saccades in SCA2 (Rodríguez-Labrada et al., 2011; Pretegiani et al., 2018). SCA2 patients may not have typical gaze-evoked nystagmus of cerebellar disease in relation to impaired ability to produce saccadic corrective phases (Geiner et al., 2008). In case of a few SCA2 patients with nystagmus starting from childhood, development of visual acuity was dependent on the amount of the foveation time. However, most SCAs with acquired nystagmus in adulthood showed well preserved vision if retinal or optic nerve abnormalities were absent.

Retinal degeneration is not usually considered part of the SCA2 phenotype. However, it has been reported that the two cases of the rare variant of SCA2 were associated with pigmentary retinal degeneration or retinitis pigmentosa (Rufa et al., 2002; Di Fabio et al., 2012).

Despite the prevalence of optic atrophy is unknown, pale optic nerve head have been reported in the majority of the SCA2 patients (Pogacar et al., 1978). Average RNFL thickness was shown to be reduced in patients with SCA2 and SCA3 (Pula et al., 2011). Scale for the Assessment and Rating of Ataxia (SARA score) reflecting severity of disease correlated inversely with RNFL thickness in SCA2.

## Spinocerebellar Ataxia Type 3

SCA3 is caused by an expansion of the CAG repeat within the *ATXN3* gene on chromosome 14q32.12. SCA3 encodes the protein ataxin-3, an ubiquitin-binding protein, probably involved in the proteasomal protein degradation pathway (Buttner et al., 1998; Chai et al., 2004).

SCA3, synonymous with Machado–Joseph disease (MJD), is one of the most prevalent autosomal dominant cerebellar ataxias worldwide, comprising approximately 20% of all SCAs. The average age of onset is usually 40s presenting with prominent cerebellar signs. A mild form demonstrated sleep disturbances and peripheral neuropathy, on the other hand, a severe form can appear with rigidity and dystonia. The longer the SCA repeats were, the earlier was the onset of SCA3, especially in men (Sequeiros and Coutinho, 1993). The majority of SCA3 has severe oligopontocerebellar degeneration, and atrophy of spinocerebellar tracts and also can present with moderate atrophy of the subthalamic nucleus, substantia nigra, internal globus pallidus, dentate nucleus, and pontine nuclei (Seidel et al., 2012).

Saccadic dysmetria with rebound and gaze-evoked nystagmus result from cerebellar dysfunction in SCA3/MJD. Saccades can be slow, and impairment of steady fixation with saccadic intrusions, including square wave jerks have frequently been reported (Kim et al., 2013; Moscovich et al., 2015). Nystagmus was common and can be considered an early ocular manifestation in SCA3 before onset of gait disturbance (Raposo et al., 2014). Complete ophthalmoplegia or gaze paralysis have rarely been reported (Stephen and Schmahmann, 2019). SCA3/MJD unlike SCA1 or SCA2 also demonstrate impaired vestibulo-ocular reflex (VOR) gain or vestibular are flexia that reflects pathologic involvement of the vestibular nuclei in the lateral brainstem apart from the cerebellum (Buttner et al., 1998).

It has been reported that SCA3 can show reduced thickness in the macular region (Pula et al., 2011), but there is no report of structural retinal abnormalities in SCA3 patients using OCT. Alvarez et al. reported that nine patients with SCA3 showed average best corrected visual acuity of 20/25, ranging from 20/30 to 20/20 at the last follow-up visit and the color vision was preserved (Alvarez et al., 2013).

Optic atrophy may be seen on fundus examination in patients with SCA3. However, either CAG repeat length or disease duration were not correlated with the optic atrophy. Recent reports found that average RNFL thickness of patients with SCA3 was thinner than that of the general population and a tendency of mild thinning of RNFL in SCA3 patients was also shown (Jardim et al., 2001; Pula et al., 2011; Alvarez et al., 2013). However, the reduction in RNFL thickness was most severely affected in the superior and inferior quadrants, and it was preserved in the temporal quadrant, indicating that the papillomacular bundle and visual acuity were well preserved.

RNFL thickness correlated inversely with ataxia rating scale scores, which implies RNFL thickness could be considered as a biomarker of the disease severity (Alvarez et al., 2013). On the other hand, no significant association between RNFL thickness and disease duration was observed (Jardim et al., 2001; Spina Tensini et al., 2017).

## Spinocerebellar Ataxia Type 6

SCA6 is caused by an expansion of CAG repeat tract within the *CACNA1A* gene located on chromosome 19p13 and is the only polyQ disease caused by mutations in a membrane protein (Vigues et al., 1999; Mondal et al., 2013).

SCA6 is distinguished from the other common SCAs in that it exhibits a pure cerebellar cortical syndrome without brainstem involvement and extracerebellar clinical manifestations (Rentiya et al., 2020). Only SCA6 among autosomal dominant SCAs belongs to ADCA type 3. The average age of onset is typically in the 50s and SCA6 has a relatively normal life expectancy. Age of onset inversely correlates with the expanded CAG repeat number.

There is a distinct feature of oculomotor abnormalities in SCA6 induced by isolated cerebellar pathology, consisting of severely impaired smooth pursuit gain, saccadic dysmetria, horizontal and vertical nystagmus, and impaired VOR gain function (Kim et al., 2013). Eye movement abnormalities including impaired saccade velocity, saccadic dysmetria and pursuit gain are the earliest functional deficits in SCA6 before the onset of ataxia (Christova et al., 2008). Diverse kinds of nystagmus may be seen; however, in SCA6, spontaneous downbeat nystagmus more common than in other types of SCA (Gomez et al., 1997). Slowing of saccades and ophthalmoparesis are rare (Stephen and Schmahmann, 2019). An increased frequency of square wave jerks might be seen in presymptomatic patients with SCA6 (Moscovich et al., 2015) (Christova et al., 2008). Eye movement in primary position and square wave jerks were noted in patients with SCA6 as well as SCA2 and SCA3 (Stephen and Schmahmann, 2019).

It has been reported that SCA6 can show reduced central macular thickness and RNFL thickness (Pula et al., 2011). However, there is no report of definite structural or functional abnormalities in the retina and optic nerve using multimodal exams in SCA6; thus, it was named pure cerebellar ataxia.

## Spinocerebellar Ataxia Type 7

SCA7 is a rare disease induced by expansion of CAG repeats in the coding region of the *ATXN7* gene located on chromosome 3p14.1, leading to the expression of ataxin-7 with an abnormal polyQ tract (David et al., 1997). While the normal range of CAG repeats is 34 and below, pathological alleles contain more than 35 repeats (Garden and La Spada, 2008). It is thought that these polyQ expansions induce dual effects: one is loss of the physiologic original protein function and the other is toxic gain of function to the mutant protein (Zoghbi and Orr, 2000). The longer the polyQ expansion, the more severe are the symptoms and the earlier is the disease. SCA7 family studies demonstrate that the CAG length show a tendency to expand through generations that induce decreasing age of onset and increasing severity with successive generations (Garden and La Spada, 2008).

SCA7, caused by a polyQ expansion in ataxin-7, is the only SCA belonging to ADCA type 2 characterized by progressive cerebellar ataxia and concomitant retinal degeneration (Sun et al., 2016). The spinocerebellar degeneration, atrophy of pyramidal tracts and motor nuclei in the brainstem, retinal degeneration, and ataxin-7 immunoreactive neuronal intranuclear inclusion are involved in the pathology of SCA7 (Martin, 2012).

The initial presenting sign of SCA7 is typically gait ataxia that can progress over the following years. Additional neurologic manifestations include ophthalmoplegia, slow saccadic movements, dysphagia, dysarthria, pyramidal tract signs, and parkinsonism in particular.

Oculomotor abnormalities may include saccadic pursuits, saccadic dysmetria, slowed saccades and gaze-evoked nystagmus (Schöls et al., 2008), progressing to ophthalmoparesis (Teive et al., 2012). These relatively non-specific signs are commonly found in the olivopontocerebellar atrophies. When combined with the retinal abnormalities, the oculomotor signs can provide a specific diagnosis. Particularly in SCA7 and SCA2, saccadic slowing with prolonged latency was common and present even in early stage, possibly leading to ophthalmoplegia in late stage (Oh et al., 2001; Federighi et al., 2011; Stephen and Schmahmann, 2019).

What distinguishes SCA7 from other SCAs is that it is associated with progressive visual loss. Horton et al. found that 9 of 13 patients with symptomatic SCA7 complained of visual disturbances, with vision ranging from 20/30 to light perception (Horton et al., 2013). Onset of visual loss may occur in childhood and visual disturbances were affected by the degree of degeneration of the retina or macula. In patients with SCA7, visual disturbances may precede, appear together, or follow the onset of the ataxic symptoms (Michalik et al., 2004). Thus, of all the SCAs, the role of the ophthalmologist in the diagnosis and management is paramount in SCA7. This retinal degeneration initially involves cones but progresses to affect the entire retina, leading to a cone-rode dystrophy and blindness (Michalik et al., 2004).

**FIGURE 1:**
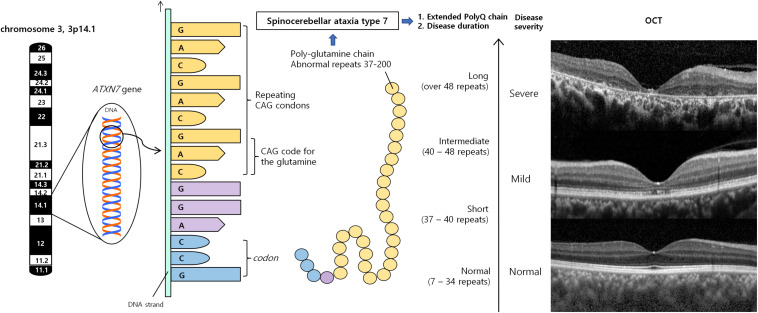
Spinocerebellar ataxia type 7 results from an expanded polyglutamine tract, encoded by CAG repeats. Misfolded polyQ proteins form aggregates which lead to various cellular process dysfunctions, leading to cell toxicity and degeneration. Retina is the one of the most severely affected region. According to number of repeats and duration of diseases, retinal manifestations can vary from no abnormality, occult macular dystrophy, cone-rod dystrophy to severe retinal degeneration. OCT demonstrated substantial diffuse macular thinning, especially outer retina including photoreceptor at fovea, and disorganization of all retinal layer, corresponding to severe macular degeneration, which can be seen at late stage of cone-rod dystrophy (upper), mild foveal thinning, and foveal photoreceptor outer segment disruption, which can be seen occult macular dystrophy or very early stage of cone-rod dystrophy (middle), and normal retina (lower). CAG, cytosine–adenine–guanine; OCT, optical coherence tomography; *PolyQ*, polyglutamine proteins.

SCA7 is the only SCA that almost always has progressive retinal degeneration. The degree of retinal degeneration may vary depending on the stage of the disease course and the number of CAG repeats in the ataxin-7 gene (Figure 1). In almost all SCA7 patients, visual acuity was decreased at the first visit, ranging from 20/40 to light perception (Manrique et al., 2009; Park et al., 2019).

Especially at the initial stage of the course of the disease, fundus can appear normal. Subtle changes such as blunting of the normal foveal reflex, fine granular pigmentary macular changes or retinal arterial attenuation can be discovered through precise inspection (Thurtell et al., 2009).

In case of the CAG repeats count is low (< 40 repeats), fundus can appear normal and full-field ERGs showed normal response both in dark- and light-adaptation. However, spectral-domain OCT can find focal disruptions in the photoreceptor ellipsoid zone and the photoreceptor outer segment–retinal pigment epithelium interdigitation zone and multifocal ERGs demonstrate reduction of central responses. Thus, SCA7 can present phenotypes similar to the occult macular dystrophy in case of the lower number of CAG repeats (Park et al., 2019).

Generally, however, SCA7 can present retinal degeneration similar to cone-rod dystrophy (Drack et al., 1992; Aleman et al., 2002). Experimental Studies have demonstrated that polyQ expanded ataxin-7 disrupts the action of the cone-rod homeobox (*CRX*) gene, a transcription factor regulating photoreceptor genes (La Spada et al., 2001). One of the causative genes in cone-rod dystrophies is *CRX* mutations. Inappropriate interactions between expanded *ataxin-7* and *CRX* are known to cause the phenotype similar to cone-rod dystrophy (Aleman et al., 2002).

Funduscopic findings can vary from minimal granular retinal pigment epithelial changes, vascular attenuation, or atrophy of the macula and optic nerve, to a bull’s-eye maculopathy, demonstrated in patients with cone–rod dystrophy found in SCA. Relatively equal reduction of cones and rods is shown in full-field ERG responses. Central and peripheral retinal regions are more severely affected and showed greater dysfunction than the midperiphery. Multifocal ERG demonstrated predominantly central involvement, especially in the early disease stages, with progression extending from the center to the more peripheral retinal areas (Hugosson et al., 2009).

With disease progression, vascular attenuation, optic disk pallor, prominent granular pigmentation, retinitis pigmentosa-like diffuse pale and pigmentary atrophy could be observed (Ahn et al., 2005; Levinson et al., 2016). However, concurrent neurologic signs and oculomotor abnormalities with relatively rapid progression can help differential diagnosis between RP and SCA7 initially. Full-field ERG in the patients with progression indicated distinctly prolonged 30-Hz flicker implicit time and loss of cone and rod responses, verifying widespread photoreceptor degeneration.

Macular and retinal dysfunction may be diagnosed via multifocal ERG or OCT before the funduscopic abnormalities appear. OCT shows thinning of the macula and RNFL, and various degrees of atrophy of the inner and outer nuclear and plexiform layers, photoreceptors, and retinal pigment epithelium (Levinson et al., 2016; Azevedo et al., 2019). Corresponding to OCT findings, histology also demonstrated the severe loss of both photoreceptors and ganglion cell neurons, and thinning of all retinal layer including nuclear and plexiform layers, with the migration of pigment from the retinal pigment epithelium to the atrophic area (Michalik et al., 2004). Azevedo et al. suggested that visual fields, macular OCT, and Spinocerebellar Ataxia Functional Index (SCAFI) can be a candidate for state biomarkers for SCA7 starting from pre-symptomatic phases (Azevedo et al., 2019).

Color vision impairment, especially blue–yellow color vision defects, with blurred vision may occur years before other neurologic manifestations develop (Michalik et al., 2004; Miller et al., 2009). Pupillary light reactions may be slow due to the loss of retinal ganglion cells.

In SCA7, ataxin-7 is known as an element of the multi-protein Spt-Ada-Gen5 acetyltransferase (SAGA) complex that plays a role as coactivator for RNA polymerase II transcription (Bonnet et al., 2014). Mutant ataxin-7 alters SAGA activities, following its integration in the complex or by sequestration of the SAGA components in insoluble nuclear inclusions, leading to transcription deregulation. Moreover, mutant ataxin-7 is associated with the impairment of the ubiquitin–proteasome process and autophagy system, responsible for the clearance and degradation of misfolded proteins, resulting in abnormal protein aggregation, which leads to form the nuclear inclusions over time. It is also known to affect the mitochondria function, apoptosis, and the differentiation of photoreceptors and Purkinje neuronal cell in SCA7 (Yoo et al., 2003; Mookerjee et al., 2009; Wang and Dent, 2014). In SCA7 knockin and transgenic mouse models, misfolded mutant ataxin-7 accumulates more quickly in the nucleus of vulnerable neuronal cell types such as photoreceptors and Purkinje cells, showing a close relationship between accumulation and toxicity (Yvert et al., 2000).

A few experimental studies have led to the specific hypothesis that the retinal phenotype in SCA7 can be seen as a result of interference with the action of the *CRX* gene, expressed predominantly in retinal photoreceptor cells and controlling the expression level of multiple photoreceptor-specific genes, including rhodopsin and the color opsins (La Spada et al., 2001). In the R7E transgenic mouse model, which expresses mutant ataxin-7 toxicity only in rods, R7E rods primarily lose their outer segment and progressively relapse to round cell shape, consistent with the loss of photoreceptor differentiation identity (Yefimova et al., 2010). It has been postulated that retinal degeneration in SCA7 may be associated with an abnormal interaction between the expanded ataxin-7 and *CRX* as potentially binding the expanded polyQ tract in ataxin-7 to a glutamine-rich region in *CRX* (La Spada et al., 2001; Yoo et al., 2003; Kumar and Pulido, 2011). During the developmental process of zebrafish, ATXN7 inactivation induces diminished expression of *CRX* and rhodopsin genes that leads to malformation of the outer segment of the photoreceptor (Yanicostas et al., 2012; Carrillo-Rosas et al., 2019). It is postulated that one of the primary toxic effects of polyQ expansion is to induce the altered function of human *ATXN7* in terms of maintaining differentiated photoreceptors (Abou-Sleymane et al., 2006). Recently, in the SCA7 R7E transgenic mouse model, Lebon et al. has reported that molecular pathway of photoreceptor cell death is related to a caspase-independent apoptosis induced by consequences of ataxin-7 polyQ protein expression in the retina. They have shown that polyQ ataxin7 activates muller cells, endoplasmic reticulum stress accompanied by an unfolded protein response, and cell death effectors, such as Apoptosis-inducing Factor and Leukocyte Elastase Inhibitor, resulting in photoreceptor cell loss (Yefimova et al., 2010; Leprêtre et al., 2013; Lebon et al., 2019).

It has been reported that SCA7 can show reduced peripapillary RNFL thickness; however, the temporal quadrant was preserved except in advanced disease (Manrique et al., 2009; Campos-Romo et al., 2018). Campos-Romo A et al. have demonstrated that optic nerve analysis of 16 SCA7 patients having CAG repeats ranging from 42 to 61 showed normal ranges for disk area, cup area, neuroretinal rim area, and cup/disk ratio and average RNFL thickness, except for RNFL thinning, in only 2 subjects (Campos-Romo et al., 2018).

The previous study reported a reduction in corneal endothelial cells and increased corneal thickness (Manrique et al., 2009), although the corneas remained clear, and the decreased endothelial cell density showed inverse correlation with the severity of the motor symptoms in patients with SCA7. The mechanisms are yet unknown (Campos-Romo et al., 2018).

## Spinocerebellar Ataxia Type 17

SCA17 is one of the autosomal dominant cerebellar ataxia by abnormal CAG/CAA repeat expansion encoding a polyQ tract in the TATA-box binding protein (TBP) gene on chromosome 6q27 (Liu et al., 2019). TBP is a transcription factor essential for formation of the transcription preinitiation complex and transcription of RNA polymerase I, II and III (Burley, 1996). Inactivation of TBP induces downregulation of transcription, growth arrest and cell death (Martianov et al., 2002). Furthermore, polyQ-expanded TBP fragments, which were incapable of binding DNA, formed nuclear inclusions and caused a neurodegeneration (Friedman et al., 2008). Normal alleles contain 25 to 40 CAG/CAA repeats. The number of pathologic repetition is 43 or more. However, 41 through 43 repeats should be considered as the intermediate range, requiring cautious interpretation (Shin et al., 2015; Origone et al., 2018). Almost all patients have a family history and the disease is inherited as an autosomal dominant trait. However, a few cases develop *de novo* mutations in the TBP gene (Shatunov et al., 2004). Clinical variability is high, even in the same families or in individuals with the same size of expansions (Koide et al., 1999).

Ataxia and/or dementia are main clinical symptoms in SCA17 patients and observed as an initial symptom (Toyoshima and Takahashi, 2018). Dementia is the second most common symptom throughout the disease course. Behavior or personality alterations and psychiatric symptoms, including euphoria, depression and aggression, are observed frequently (Mariotti et al., 2007). Clinical manifestations similar to Huntington disease, characterized by chorea, parkinsonism, and psychiatric features or dementia, are often observed in patients with large trinucleotide repeats (Koutsis et al., 2014). Furthermore, epilepsy and extrapyramidal signs such as dystonia, and akinesia may be also present (De Michele et al., 2003; Rolfs et al., 2003).

Oculomotor abnormalities have often been reported (Rosini et al., 2020). Normal saccade velocity, with saccadic hypometria was observed in SCA17 patients (Hübner et al., 2007). Smooth pursuit initiation and maintenance were affected, with increased latency and decreased acceleration. Gaze-evoked, rebound, and downbeat nystagmus can be present. There is a report on anti-saccades and memory-guided saccades demonstrating significant increased error rates in SCA17 patients (Mariotti et al., 2007) (Hübner et al., 2007). Some SCA patients can show hyperreflexia of VOR with absence of nystagmus (Mariotti et al., 2007). These oculomotor abnormalities were related to disease duration, but not to number of repeats.

There is no report of definite structural or functional retinal and optic nerve abnormalities using multimodal exams in SCA17.

## Therapeutic Strategies for Ophthalmic Abnormalities

Probably the most comprehensive review of possible drug targets of patients with SCAs was performed by Lazlo et al. in 2019 (Szpisjak et al., 2019). The detailed review of the molecular targets and therapeutic strategies of each SCAs have recently been reviewed elsewhere (Perez Ortiz and Orr, 2018; Egorova and Bezprozvanny, 2019; Ishikawa and Nagai, 2019; Niewiadomska-Cimicka and Trottier, 2019). Therefore, the objective of this section is to give a brief description of the main therapeutic options in terms of ophthalmic pathology in the most common polyQ SCAs (SCA1, 2, 3, 6, and 7).

There is currently no approved preventive or curative treatment for ophthalmic abnormalities including retinal degenerations in SCA patients. Although many molecules, such as ceftriaxone for reducing glutamate levels (Lee et al., 2008; Maltecca et al., 2015), hepatocyte growth factor for neurotrophic effects (Ieraci et al., 2002), and ion channel modulators including riluzole (Romano et al., 2015) and valproic acid (Lei et al., 2016), may have a potential to improve the ophthalmic symptoms and protect retinal and optic nerve degeneration, no definite evidence of benefit was established. Recently, gene silencing techniques including RNA interference (RNAi) and antisense oligonucleotides (ASO) may consider one of the options for the treatment of polyQ disorders, which have been validated in several mouse models. The RNAi, using short double-stranded RNA designed to trigger degradation or translation repression of sequence-specific homologous RNA targets, within cells of the central nervous system and retina has demonstrated potential to have therapeutic effect in animal models of repeat expansion SCAs including SCA1 (Xia et al., 2004; Keiser et al., 2013, 2014, 2015), SC3/MJD (Alves et al., 2010; Durcan et al., 2011; Costa Mdo et al., 2013; Rodriguez-Lebron et al., 2013), SCA6 (Miyazaki et al., 2016), and SCA7 (Ramachandran et al., 2014a,b). A previous preclinical study has reported no adverse toxicity and a preservation of normal retinal function with more than half reduction of mutant and wild-type ataxin-7 allele through the subretinally injected RNAi technique (Ramachandran et al., 2014a).

Similarly, ASOs, single-stranded chemically modified nucleic acids to prevent the translation, and mediate the destruction that target specific SCA genes and modify the processing have turned out to be effective in animal models of several SCAs, such as SCA2 (Scoles and Pulst, 2018), SCA3/MJD (McLoughlin et al., 2018), and SCA7 (Niu et al., 2018). Preclinical studies have found that in SCA7 mice with retinal disease, visual function was improved after intravitreal injection of ataxin-7 ASOs even though initiating treatment after symptoms have occurred (Niu et al., 2018).

Such treatments can be applied from early stages of the disease in patients with polyQ SCAs, even before symptoms begin. Furthermore, because of the possibility of intraocular injections to deliver the vector close to the target retina, quantitative evaluations for monitoring therapeutic responses through OCT and electrophysiologic testing, and the rapid disease progression in SCA7, RNAi and ASOs targeting ataxin-7 may be a feasible treatment for SCA7 retinal degeneration. Excitingly, nucleotide-based gene silencing strategies for some SCAs is expected to enable active clinical trials in humans within the next few years (Klockgether et al., 2019).

## Challenges and Future Perspectives

When seeing patients who visit an ophthalmology clinic presenting with eye movement abnormalities and/or visual disturbances, SCAs can be easily misdiagnosed, because it is difficult to consider by connecting the SCAs with the ophthalmic abnormalities due to a substantial rarity, and phenotypical diversity. However, recently, various reports of SCA’s ophthalmic manifestations, multimodal imaging and various functional analysis can help ophthalmologists become familiar with SCAs and differentiate it from other neurodegenerative diseases with ophthalmic abnormalities.

Although recent advances in our understanding of the pathogenesis mechanism in SCAs through biochemical research using cellular and animal models of SCAs propose promising way for both symptomatic and disease-modifying therapies, so far, any disease-modifying and systemic drugs has not proven to be effective in preventing or relieving photoreceptor degeneration or optic nerve atrophy. Much more needs to be investigated in order to clearly understand the pathogenic processes underlying neuronal specific dysfunction and following neuronal cell loss. Future research for analysis of the molecular and physiologic similarities between cerebellum and retina and to specify the neurodegenerative mechanisms of the optic nerve and retina in the presence of mutant ataxin may be necessary. Additional basic researches are also needed to identify new molecular targets for SCAs to yield medications with obvious advantages for SCAs (Egorova and Bezprozvanny, 2019).

In contrast, the therapeutic potential of many molecules remains unrevealed, because of complexity due to their intrinsic properties including poor water solubility and poor pharmacokinetic properties, and difficulty penetrating the blood–retina barrier. Therefore, there are increased needs to develop drug delivery systems, such as nanoparticles, to improve target specificity and enhance bioavailability of drugs in the retina.

In inherited retinal dystrophies caused by defective genes, gene therapy is being developed to treat the causative genetic problems and one product (Luxturna) has been approved by FDA (Apte, 2018). Eye is a fascinating target for gene therapy as local treatment is possible evading the systemic immune reactions while allowing the effective gene delivery (Singh et al., 2020). For SCA1 or SCA7, ocular gene therapy might be possible in the future fixing the genetic defect in the retinal cells. Stem cell therapy is another promising treatment for inherited retinal diseases but it has not been proven to be effective for ophthalmic diseases through clinical trials (Singh et al., 2020). If the safety issues can be overcome, stem cell therapy may also be a potential therapeutic option for retinal and optic nerve degeneration in patients with SCA. Other future therapies for ophthalmic problems from SCA could be optogenetics, genome editing, and retinal prosthesis.

The emergence of disease-modifying therapy for SCAs, including RNAi and ASOs, may give promising results in the treatment of SCAs, but the lack of sensitive biomarkers in terms of evaluation of the treatment efficacy or disease progression and the small number of patients to be recruited in clinical trials are obstacles for the clinical research. Outcome measurement, which provides information on treatment efficacy, is essential for these genetic diseases that can be treated, especially before symptoms begin (Coarelli et al., 2018). Compared to the brain, the eye can be evaluated easily and precisely with regards to the anatomical structure and physiologic function, especially the retina and optic nerve, through optical and electrophysiologic equipment including OCT, OCT angiography, full-field ERG, and multifocal ERG. Therefore, these multimodal imaging and tests have been shown to have potentials as markers of disease progression in patients with SCAs. It is possible to detect retinal diseases and optic nerve abnormalities in a more mild state at an early stage and identify the mechanisms and patterns of ophthalmic abnormalities through the development of retinal/optic nerve imaging techniques in the future. In addition, the advanced eye tracking system or ocular movement recording system with analysis software will be able to more precisely analyze oculomotor abnormalities as a potential biomarker of disease progression and effectiveness of treatment. Larger longitudinal studies will be needed to identify the role of each examination.

Further fundamental and clinical studies are required to identify the comprehensive mechanisms and qualify the long-term effects and safety of various treatments of SCAs. Nevertheless, future clinical trials using RNAi, ASO, stem cell treatment, and various molecules such as Ca^2 +^ stabilizers and ion channel modulators will be expected to provide a promising prospect for the treatment of incurable SCA and other polyQ expansion diseases.

## Conclusion

A variety of ophthalmic symptoms are seen in patients affected by SCAs and can lead to various degrees of visual loss and functional disability. The advent of ocular imaging technology, particularly OCT, has enabled the quantification of neurodegeneration by providing cross-sectional images of the retina and optic nerve. Various oculomotor abnormalities, such as saccade abnormality, nystagmus, impaired VOR, and ophthalmoplegia, can be observed according to the underlying pathology including cerebellum, cerebral cortex, brainstem, and spinal cord. RNFL thinning and/or optic atrophy has been documented in several SCA types including SCA1, SCA2, SCA3, SCA6, and SCA7. Retinal degeneration was often observed in certain types of SCAs. In SCA7, the degree of retinal degeneration may vary from normal fundus, occult macular dystrophy, cone-rod dystrophy to severe atrophy depending on when the disease course was observed and the length of CAG repeats in the ATXN7 gene. SCA1 often demonstrates relatively mild macular degeneration compared to SCA7. The level of visual impairment varies widely and ranges from asymptomatic to severe visual loss.

Multiple clinical characteristics of SCAs are associated with the visual system, and it might be the presenting sign in some cases. Comprehensive analysis of ophthalmic manifestations including not only the anatomy and function of the retina, and optic nerve but also oculomotor abnormalities could therefore help in the differential diagnosis among SCAs and other inherited neurodegenerative disorders and provide potential biomarkers for disease progression and therapeutic efficacy. The ophthalmologists, when being aware of the key ophthalmic features of SCA, can greatly contribute to the care of SCA patients in collaboration with other departments as a team.

## Author Contributions

JP and KJ performed material preparation and data collection. JP drafted the first manuscript. All authors commented on previous versions of the manuscript, contributed to the study conception and design, read and approved the final manuscript.

## Conflict of Interest

The authors declare that the research was conducted in the absence of any commercial or financial relationships that could be construed as a potential conflict of interest.
